# Event extraction of bacteria biotopes: a knowledge-intensive NLP-based approach

**DOI:** 10.1186/1471-2105-13-S11-S8

**Published:** 2012-06-26

**Authors:** Zorana Ratkovic, Wiktoria Golik, Pierre Warnier

**Affiliations:** 1 MIG INRA UR1077 Domaine de Vilvert, F-78352 Jouy-en-Josas, France; 2 LaTTiCe UMR 8094 CNRS Université Paris 3, 1 rue Maurice Arnoux, F-92120 Montrouge, France; 3 LIG - Université Joseph Fourier, 385, rue de la Bibliothèque, BP 53, F-38400 Saint-Martin-d'Hères, France

## Abstract

**Background:**

Bacteria biotopes cover a wide range of diverse habitats including animal and plant hosts, natural, medical and industrial environments. The high volume of publications in the microbiology domain provides a rich source of up-to-date information on bacteria biotopes. This information, as found in scientific articles, is expressed in natural language and is rarely available in a structured format, such as a database. This information is of great importance for fundamental research and microbiology applications (*e.g*., medicine, agronomy, food, bioenergy). The automatic extraction of this information from texts will provide a great benefit to the field.

**Methods:**

We present a new method for extracting relationships between bacteria and their locations using the Alvis framework. Recognition of bacteria and their locations was achieved using a pattern-based approach and domain lexical resources. For the detection of environment locations, we propose a new approach that combines lexical information and the syntactic-semantic analysis of corpus terms to overcome the incompleteness of lexical resources. Bacteria location relations extend over sentence borders, and we developed domain-specific rules for dealing with bacteria anaphors.

**Results:**

We participated in the BioNLP 2011 Bacteria Biotope (BB) task with the Alvis system. Official evaluation results show that it achieves the best performance of participating systems. New developments since then have increased the F-score by 4.1 points.

**Conclusions:**

We have shown that the combination of semantic analysis and domain-adapted resources is both effective and efficient for event information extraction in the bacteria biotope domain. We plan to adapt the method to deal with a larger set of location types and a large-scale scientific article corpus to enable microbiologists to integrate and use the extracted knowledge in combination with experimental data.

## Background

Scientific documents on bacteria biotopes describe the locations from which bacteria have been isolated. The amount of knowledge about bacteria and their isolation sites is rapidly growing due to the advancement of species identification technologies. A better understanding of the interaction of bacteria with their environment, phenotype, metabolism and genetics could be achieved by studying their correlations with their biotope properties. The availability of an increasing amount of biological data makes such large-scale studies possible [1]. A formal representation of the taxa associated with their locations is a first critical step. Descriptions of bacteria isolation sites that are available in public biology databases and biological resource centers are incomplete and rarely normalized [2]. Scientific papers and genome sequencing project web pages are an invaluable source of such information.

The BioNLP 2011 Bacteria Biotope shared task (BB) has defined bacteria location identification as an event extraction task. The participants are required to precisely identify bacteria and their locations in the form of text bound annotations of scientific documents. There are eight location types to be predicted: Host, Host-part, Geographical, Food, Medical, Soil, Water and Environment. Each of these eight location types must be related to a bacterium by a localization relation. Host and Host-part locations must be related by a Part-of relation as well.

**Figure 1:**
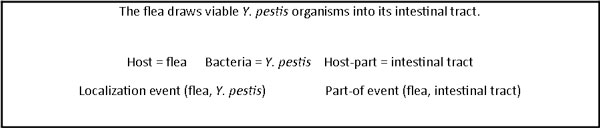
**Biotope example**. An example of a Bacteria Biotope event extraction task (BTID-50185).

The participants were provided with annotated training and development datasets. The experimental results of the participating methods, including precision, recall and F-score, as defined in [3], were measured using an independent test set. This evaluation not only measures the overall quality of the event extraction but also the recall and precision of the location arguments per type. Three teams participated in the BB task. The official results were computed in March 2011 by the organizers and published at the BioNLP workshop [4]. We realized new developments that significantly improve the results on the test set. Their evaluation was computed using the online BioNLP evaluation system and are reported here. Figure 1 provides an example of a development corpus sentence and the target biotope information. The wide range and diversity of the bacteria location vocabulary is one of the particularities of the BB task compared with other challenges on location recognition, such as protein subcellular locations in biology [5] or geographical locations in the ACE corpus [6]. There are also considerable variations in location form and structure. Thus, predictions of bacteria locations in natural language texts cannot be handled by straightforward lexicon mapping. To overcome the incompleteness of lexical resources and the corpus variability, we propose a more flexible mapping recognition method based on a symbolic syntactic and semantic analysis of both lexicon and corpus terms. This mapping filters out non-location terms and derives the type of corpus location terms along the BB task typology. We applied this method for the extraction of location names that are not denoted by rigid designators [7], *i.e*., environment (water, soil, medical), host parts and food. For the prediction of bacteria and locations that are denoted by rigid designators, *i.e*., hosts and geographical entities, we designed a lexicon-based method. We used available dictionaries along with a limited number of domain-specific morphological variation rules. Another challenge was the high frequency of bacteria anaphors and ambiguous antecedent candidates in the corpus. Our Alvis system implements an anaphora resolution algorithm that considers the anaphoric distance and the position of the antecedent in the sentence (Anaphora Resolution section). Finally, Alvis predicts the relations between the bacteria and the locations using argument co-occurrence and trigger words (Relation Extraction section).

## Methods

**Figure 2:**
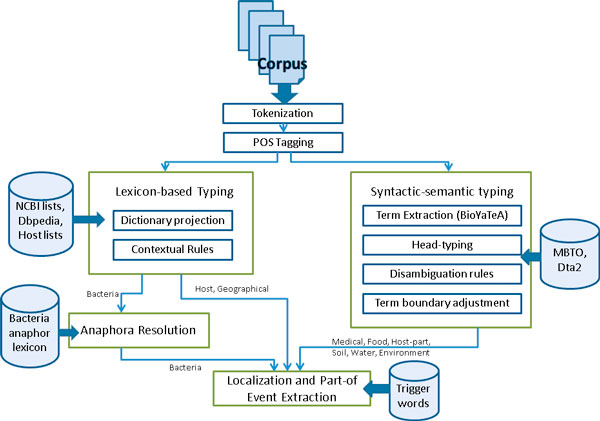
**BB event extraction workflow**. Outline of the BB event extraction workflow.

The processing steps designed for the BB event extraction task were developed using adapted Alvis pipeline modules. The Alvis pipeline is a generic NLP platform that can perform a wide variety of linguistic analyses [8]. The modules used for this task include tokenization, POS-tagging, named-entity recognition and term analysis. The Alvis pipeline was extended to include custom modules for anaphora resolution and the semantic annotation of location concepts and relations for event extraction. Figure 2 shows the outline of the workflow for the BB event extraction described below. Alvis separately handles the recognition of bacteria, host and geographical locations (Lexicon-based method section) and the recognition of environmental, medical and host-part locations (Symbolic syntactic-semantic method section).

### Lexicon-based method

A lexicon-based approach appeared more appropriate to predict bacteria, host and geographical entity names because these are predominantly proper names. Additionally, these locations are more subject to morphological variations, whereas other location names display syntactic variability.

#### Bacteria

In the training corpus, we observed that not only bacteria species names but also higher level taxa (families) and lower level taxa (strains) were annotated as event arguments. For bacteria recognition, we used the prokaryote tree of the NCBI taxonomy [9] as the main resource because it includes both scientific and common names, and it is kept up to date. We applied rewriting rules to extend it with standard abbreviations of scientific names (e.g., *Bacillus subtilis *= *B. subtilis*). It was also manually enriched by 213 bacterium taxa from the training corpus, in particular, non-standard abbreviations (e.g., *Chl*. = *Chlorobium*, *ssp*. = *subsp, GSB *for *green sulphur bacteria*) and plurals (e.g., *Vibrios *as the plural for *Vibrio*). The original NCBI taxonomy contains 270,190 bacteria names while the extended version contains 407,147.

Determining the correct boundaries of the bacteria names was a major issue because the strain names found in the corpus do not always follow conventional nomenclature rules [10]. Five specific rules were developed to account for irregular forms that could not be found by exact match. (1) Variation in nomenclature naming, such as the inclusion of the word *strain *or the abbreviations *sp*. or *spp*. (e.g., *Bordetella petrii strain DSM12804*; *Borrelia spp*.). The rule looks for both standard and non-standard nomenclature names for bacteria. (2) Variations in word order (e.g., *LB400 of Burkholderia xenovorans *instead of *Burkholderia xenovorans LB400*). This rule looks for text segments that contain all tokens of a given strain name in any order and that may contain function words, such as *of*, as in *LB400 of Burkholderia xenovorans*. (3) Parentheses (e.g., *Tropheryma whipplei (the Twist strain) *instead of *Tropheryma whipplei strain Twist*). This rule handles bacteria names with the strain name in parenthesis, which may contain other function words. (4) Partial strain names (*strain DSMZ 245 *standing for *Chlorobium limicola strain DSMZ 245 T*). (5) Bacteria names that contain modifiers such as *antimicrobial-resistant C. coli *or *L. pneumophila serogroup 1*. Such modifiers are not found in the NCBI lexicon. In the training and development sets, 107 instances of bacteria were extracted using the above rules. These five rules improve bacteria recall by 1.7 points (from 82.6% to 84.3%). Furthermore, they considerably improve the overall system recall by 13.7 points (from 38.4% to 52.1%).

The main source of error in bacteria name prediction is the ambiguity caused by the use of genus and strain name abbreviations within the same text. This error frequently occurs when the species scientific name is abbreviated using only the first word, which is also the genus name. For example, *Bartonella henselae *species is abbreviated as *Bartonella*. Unfortunately, the *Bartonella *genus is also mentioned in the same text and thus yields an ambiguity between the species anaphor and the genus name. This issue is further addressed in the Anaphora Resolution section.

#### Host locations

The same strategy was used for host name recognition. We considered eukaryote species as bacteria hosts, neglecting bacteria as possible candidate hosts. The eukaryote subtree of the NCBI taxonomy was the main source of host names (811,392), which was complemented with standard abbreviations (2,110,434). Our system uses a list of 125 common English words to remove ambiguous taxon names, such as *Indicator *(*honeyguides*) and *Dialysis (xylophages insect)*, because such terms are more frequent as common words than as taxon names. Removing these common words significantly increased the host precision by 16.6 points (from 38.8% to 55.4%). This list was enriched using 412 non-taxonomic host groups (*e.g*., herbivores), progeny names (*e.g*., calf) and human categories (*e.g*., patient). The resulting host name list contains 2,078,092 scientific names and 32,186 common names.

#### Geographical locations

At first, we considered using the very rich resource GEOnet Names Server (GNS) [11] for geographical name recognition. However, it contains too many ambiguous names to be directly usable for short-term development, as required by the challenge. For the challenge, we used the short list of Agrovoc geographical names [12]. The official recall was 29%, the worst among participant systems. The current version of the Alvis geographical name recognition component uses the country list of DBpedia [13], which has 1,201 entries and includes country and US state names. An additional contextual pattern increases the recall of the geographical name recognition, which considers the proper names preceding country or U.S. state names as geographical locations as well, for example, *Mono Lake, California*. The new developments slightly improved the overall recall by 2.4 points. However, there is still room for improvement, which can be achieved using more sophisticated patterns and larger resources.

### Symbolic syntactic-semantic method

The other location entities of types Soil, Water, Medical, Environment and Host-Part cannot be handled by lexicon-based methods only. Available lexicons are necessary resources, but they cannot account for the vocabulary variety given that a location can be any physical matter. Moreover, as observed in the corpus, these location terms are noun phrases with adjectival and noun modifiers, verbal and prepositional complements subject to deep syntactic and semantic variations. We propose a robust location entity extraction method that overcomes both the high degree of variation and the incompleteness of the lexicons. The first step extracts all candidate terms from the corpus. The second step assigns a location type to the candidate terms and eliminates the irrelevant ones. This step exploits the morpho-syntactic structure of the terms and the location types derived from the location lexicon.

#### Term extraction

For term extraction, we used Alvis with BioYaTeA [8], an extended version of YaTeA [14] that has been adapted to the biology domain. We analyzed the training dataset to set BioYaTeA parameters and adapt it to the task. BioYaTeA parameters are a set of modifiable constraints that include boundaries, morpho-syntactic patterns and domain specific post-filters. We restricted the morpho-syntactic patterns to noun phrases with adjectival modifiers (e.g., *microbial mat*). Prepositional complements in the training dataset location terms are rather rare (e.g., *breaks in the skin*), except *of *prepositional complements (e.g., *nodules of plants*). The boundaries were set to include only the *of *preposition and exclude other prepositions, such as *with *or *at*, which may yield syntactic attachment errors (e.g., *contaminating an open wound with sea water*). We thus preferred the risk of incomplete terms to incorrect prepositional attachments. BioYaTeA produces a set of candidate terms with their syntactic structure in the form of head-modifier relations (e.g., *geothermal<=M> environment<=H>*).

#### Type assignment

The type assignment allocates types to BioYaTeA terms, with respect to the location lexicon, where each entry has a location type among Soil, Water, Medical, Food, Environment and Host-Part. Head identification is a crucial point for our type assignment method. We observed that, in most cases, the head of the candidate location compound conveys the location type information. However, some terms have ambiguous heads with respect to the location types (i.e., *canal *as Host-Part or Water) or uninformative heads, *i.e*., those that convey little or no type information, such as *area *or *sample*.

The type assignment algorithm considers five different cases. (1) The trivial case, the algorithm finds an exact match with a lexicon term, which generates the type of the candidate term (Table 1, example 1). (2) The candidate term is assigned the same type as the lexicon terms that have the same head (Table 1, example 2). (3) If the candidate term has the same head as lexicon terms of *different *types, then the correct type is selected by a set of disambiguation rules (Table 1, example 3). The most frequent ambiguity is the confusion Host, Environment and Food types. This reflects the different status of animals and plants with respect to bacterial contamination. Disambiguation rules take into account the context and the modifiers of the candidate term. For instance, if a term of type Host is preceded by a death-related adjective, then its type is renamed as Environment (Table 2, example 1). (4) The head of the candidate term belongs to a list of uninformative heads. The method searches for the largest subterm, *i.e*., the largest term constituent, with a relevant head (Table 1, example 4). If no such head is found, the term is not tagged as a location. (5) In the case, where none of the matching rules apply, the candidate term is not tagged as a location term (Table 1, example 5).

**Table 1:** Examples of type assignment.

	Corpus term	Matching rule	Head	Assigned Type
Ex. 1	*aquatic sediment*	Exact term	*sediment*	Environment
Ex. 2	*sludge of a zinc decantation tank*	Term head	*sludge*	Environment
Ex. 3	*tooth canal*	Term head and disambiguation	*canal*	Host-Part (not Water)
Ex. 4	*marine environment*	Subterm head	*marine *(*environment *is an uninformative head)	Water
Ex. 5	*wound infections*	None	*infection*	None

**Table 2:** Examples of disambiguation and location boundary adjustment rules.

	Rule role	Rule example	Location term
*Ex. 1*	Type disambiguation	IF *dead, decaying *in term THEN type =Env	*dead animal *(Host) becomes *dead animal *(Env)
*Ex. 2*	Boundary extension	IF nationality before term (Env) THEN include nationality	*oil field *(Env) becomes *German oil field *(Env)
*Ex. 3*	Boundary reduction	IF irrelevant modifier in term THEN delete modifier	*infected rodent *(Host) becomes *rodent *(Host)

#### Adjustment of term boundaries

The BB task guidelines specify that the location span should exclude information that is irrelevant to bacteria living conditions, such as *infected *in *infected tick*. The method adjusts the location boundaries by removing irrelevant modifiers from the extracted terms. We manually built a list of these modifiers by examining the training set, the habitat and isolation site fields of the GOLD database [15].

The guidelines also specify that location spans do not overlap. However, it is possible that the location terms predicted by Alvis are nested because BioYaTeA extracts not only the complete terms, but also their subterms (*i.e*., constituents). To choose the right subterm, the method selects the maximum disjoint terms among the extracted location terms. For instance, the term *human gastrointestinal tract *has four location subterms: *gastrointestinal tract*, *human*, *gastrointestinal *and *tract*. The maximum disjoint location terms are *gastrointestinal tract *and *human*. The others are discarded because they are overlapping.

#### Resources for type assignment

The location lexicon is processed before location prediction to compute its term heads and types. The term head is computed by BioYaTeA. The assignment strategy of types to lexicon entries depends on the structure of the lexicon, more precisely whether it is flat or hierarchical. We experimented with three different resources: the flat list of all tagged location terms of the training and development sets (.a2 files) referred to a DTa2, the Microorganism Biotope Termino-Ontology (MBTO) and the EnvO habitat ontology [2]. MBTO focuses on bacteria biotopes and phenotypes with a strong emphasis on the physico-chemical properties of the habitats. MBTO had previously been developed at the Institut National de la Recherche Agronomique (INRA). EnvO includes eukaryote habitats and is less focused on bacteria habitat descriptions.

DTa2 location terms of the task corpus are already annotated with types. To associate MBTO and EnvO concepts with the BB task types, we took advantage of the hierarchical structure of the ontologies. We manually associated the high level nodes of the location hierarchies to the eight location types. The types of the lower level concepts were then inferred. For instance, the concept *aquatic environment *is tagged as Water in the ontology, and all of its descendants, including *lake*, *sea *and *ocean*, are consequently of the same type. Local exceptions were manually handled. Once the ontologies have been processed, they can be used by the corpus type assignment algorithm.

#### Experiments with the typing method

Here, we report the experimental results of the typing method on the BioNLP BB test set. BioYaTeA extracted 1,873 candidate terms from the test set. Table 3 details the number of candidate terms typed as location terms using the three different resources. Depending on the lexicon, the method assigned location types to 9 to 16% of the test terms. MBTO yields more location predictions. However, it is also the resource with the most ambiguous heads. For all three resources, symbolic syntactic-semantic matching notably increases the number of predicted locations compared with exact match. This difference is especially marked for EnvO, for which it predicts 91% of all of the predicted locations. The evaluation of the prediction quality is detailed in the Results section.

**Table 3:** Number of terms in the test corpus per type assignment method and per resource.

	MBTO	EnvO	DTa2
% of corpus terms	16%	9%	10%
Exact match	147	46	72
Main head of term	133	114	103
Subterm head	5	4	3
Ambiguous head	26	21	17
Total head matching	164 (52%)	139 (91%)	123 (63%)

Total	311	185	195

### Related work

There are a broad range of methods that aim to generate semantic annotations of entities using types and ontologies. Among them, our type assignment method belongs to the class of non-statistical terminological methods that rely on partial parsing and the semantic analysis of terms [16]. It is strongly related to the head-similarity analysis, as previously described [17]. The way our method associates corpus terms to lexicon entries is similar to MetaMap [18]. MetaMap tags biomedical corpora with the UMLS Metathesaurus using syntactic analysis, which takes into account lexical heads of terms for matching lexicon-corpus terms. However, as opposed to MetaMap, the aim of our lexicon-corpus term matching is not to identify the semantically closer terms but to use the matching result to infer the relevant type of the corpus terms at a coarse grain level. Therefore, a resource less extensive than UMLS is required, which is indicated by the favorable results obtained using DTa2 (Results section). This matching does not have to be as precise, and consequently, the computation of term variation, as performed by MetaMap, is not necessary.

### Relation extraction

The extraction of relations from text has led to a large amount of work involving an increasing amount of linguistic analysis, including syntactic analysis, such as dependency parsing [19] and discriminant classification machine learning methods, *e.g*., ILP [20], SVM [21-23], KNN with weak supervision [24] or maximum entropy [25].

For the prediction of localization and Part-of events, we used a straightforward and efficient strategy based on the co-occurrence of arguments and trigger words within a sentence, as previously described [26]. The system predicts a localization event between a bacterium and a location when a bacteria name (or a bacteria anaphor), a location and a trigger word occur together in the same sentence. Similarly, the system predicts a Part-of event between the host and the host part when a host name, a host part term, a bacterium's name (or a bacteria anaphora) and a trigger word occur together in the same sentence. Requiring the occurrence of a bacterium and a trigger for predicting a Part-of event may appear superfluous. However, removing the bacterium's name and trigger word co-occurrence constraint negatively affects the precision of the Part-of event extraction without improving the recall. The trigger words denote the bacteria residence (e.g., *inhabit*, *lives*, *niche*, *environment*), its dissemination and contamination means (e.g., *colonize, ingest*), its pathogenic effects (*e.g*., *chronic*, *disease*, *pathogen*) and bacteria sampling (e.g., *discover*, *isolate*). The trigger word list was designed by automatically ranking words in the training corpus sentences that contain both a bacteria name and a location term. The ranking criterion used was the information gain with respect to whether the sentence actually contained an event. The ranked list was manually adjusted by removing words that do not designate a location relation and adding 35 domain knowledge words (e.g., *outbreak*, *flora, epidemic*). In total, 55 trigger words were collected. The list contains word stems to account for morphological variations and variations in part of speech categories among the triggers. To measure the potential negative effect of trigger words on the recall of the localization event extraction, we tested the algorithm without their use (Results section).

### Anaphora resolution

Anaphors are encountered frequently in the BB task corpus, especially for bacteria and to a much smaller extent for hosts. Our effort focused on bacteria anaphora resolution. The location relation extraction method, as described above, assumes that the relation arguments (location and bacterium, or anaphor of the bacterium) occur in the same sentence. From a total of 2,296 sentences in the training corpus, only 363 sentences contained both a location and an explicit bacterium, while 574 mention only a location. Thus, anaphora resolution is critical for location event extraction.

The style of some of the documents is rather relaxed, and the antecedent may be ambiguous even for a human reader. A manual examination of the training corpus indicated that the most frequent bacterial anaphors are not pronouns, but hyperonym definite expressions, either higher level taxa often preceded by a demonstrative determinant (*This bacteria, This Clostridium*) or sortal anaphors (*genus, organism*, *species, strain*). Both types of anaphors are commonly found in biology texts [27]. For anaphora detection and resolution, a pattern-based approach was preferred to machine learning because the constraints for relating anaphors to antecedent candidates of the same taxonomy level were mainly semantic and domain-dependent. Additionally, the annotation of anaphors was not provided in the training corpus.

In general, the resolution of anaphors has three steps: (1) the identification of the anaphoric expression, (2) the search for the set of possible antecedents and (3) the selection of the antecedent from the set [28]. In our case, the set of possible antecedents is computed beforehand because only bacteria anaphors are handled and the bacteria mentions are detected prior to anaphora resolution. Thus, the Alvis anaphora resolution is only required to perform the first and final steps.

We handled three types of anaphors in the corpus: standard anaphors that have a unique bacteria name as an antecedent (example 1), *bi-anaphors *that have two bacterial names as antecedents (example 2) and a higher taxon name being used as an anaphoric expression to refer to a lower taxon, which we named *name taxon anaphors *(example 3).

#### Example 1: Anaphor with a unique antecedent (BTID-60106)

***C. coli ****is pathogenic in animals and humans. People usually get infected by eating poultry that contained **the bacteria**, eating raw food, drinking raw milk, and drinking bottle water [...]*.

#### Example 2: Anaphor with two antecedents (BTID-60106)

***C. coli ****is usually found hand in hand with its bacteria relative, **C. jejuni**. **These two organisms **are recognized as the two most leading causes of acute inflammation of intestine in the United States and other nations*.

#### Example 3: Name taxon anaphor (BTID-10090)

*Ticks become infected with ****Borrelia duttonii ****while feeding on an infected rodent*. ***Borrelia ****then multiplies rapidly, causing a generalized infection throughout the tick*.

#### Example 4: Localization sentence without anaphor (BTID-60051)

*In the 1600s **anthrax **was known as the "Black bane" and killed over 60,000 cows*. The first step automatically identifies potential anaphors in the corpus given a list of pronouns, sortal anaphors and taxa [29,30].

The final step selects the most likely bacteria antecedent from the set of possible candidates to relate it to the anaphor. Much of the work performed on anaphora resolution describes the different morphological features of the anaphor/antecedent pair to match them [30]. These features include gender and number, which are useful in narrowing down the antecedent set. In our case, these features would not be useful because we are only treating bacteria anaphors and the word *bacteria *is often used to refer to both a single bacterium and the species. Therefore, we focus on two other features: the distance between the anaphor and its antecedent and the salience of the antecedent candidate in the sentence.

The antecedent is usually found close to the anaphor to maintain the coherence of the discourse. Therefore, our method ranks the antecedent candidates according to the anaphoric distance counted in the number of sentences. The closer the antecedent is to the anaphor, the more likely it is to be selected. If more than one bacterium is found in a given sentence, their position is a discriminant. Centering theory states that in a sentence the most prominent entities and, therefore, the most probable antecedent candidates are in the order: subject > object > other position [31]. In English, due to the SVO order of the language, the subject is most often found at the beginning of the sentence, followed by the object and then the others. Therefore, the method retains the leftmost bacterium in the sentence when searching for the best antecedent candidate. For anaphors that require two antecedents, we use these same criteria but search for two bacteria in each sentence or paragraph instead of one. For taxon anaphora, we search for the presence of a lower taxon in the document found before the anaphor that is compatible according to the species taxonomy.

The counts of anaphors detected by the method per corpus and per kind of antecedent are given in Table 4. It is worthwhile to note that taxon anaphors account for 12% of the total.

**Table 4:** Number of anaphors found in each corpus per anaphor type.

Corpus	Single ante	Bi ante	Taxon ante
Train	933	4	129
Dev	204	3	22
Test	240	0	18
Total	1377	7	169

The anaphora resolution algorithm allowed us to retrieve more sentences that contain both a bacterium and a location. Of the 574 sentences that contain only a location, 436 (76%) were found to also contain an anaphor related to at least one bacterium. The remaining 138 sentences are cases where there is no bacteria anaphor or the bacterium mention is implicit. The bacterium is frequently referred to through its action. For instance, in example 4 above, the anthrax bacterium name could be derived from the name of the disease that it causes.

## Results

Tables 5 and 6 summarize the scores that the Alvis system achieved on the Bacteria Biotope task. They give the scores obtained by the best configuration of the Alvis system and the improvement compared to the official scores (in parenthesis) achieved during the challenge in March 2011. The Alvis system ranked first among three participants. The new scores were computed using the online evaluation system of the BioNLP website. Table 5 shows the precision, recall and F-score of the event extraction. Table 6 details the recall of entity prediction per type.

**Table 5:** Alvis system scores for event extraction on the BB task.

	Event recall	Event precision	F-score
Part-of event	45 (23) +22	66 (79) -13	53 (36) +17
**Total**	**52 (45) +7**	**46 (45) +1**	**49 (45) +4**

The Part-of event and the total system scores are presented, as provided by the task organizers. The current system scores are found on the left while previous (BioNLP 2011 challenge) scores on the BB task are in parenthesis. The difference (+/-) between both is on the right.

**Table 6:** Alvis system scores for entity recall, recall, precision and F-score on the BB task by location type.

	Bacteria	Host	Host-part	**Env**.	**Geo**.	Water	Soil
**Entity**	84 (84)	82 (82)	76 (72)	61 (53)	70 (29)	64 (83)	88 (86)
**Recall**							
**Recall**		63 (61)	56 (53)	41 (29)	31 (13)	35 (60)	70 (69)
**Precision**		55 (48)	40 (42)	24 (24)	36 (38)	44 (55)	70 (59)
**F-score**		59 (53)	47 (47)	31 (26)	34 (19)	39 (57)	70 (63)

The current system scores are found on the left while previous (BioNLP 2011 challenge) scores are in the parenthesis. Medical is not included because it is not significant. Food was not found in the test corpus.

The overall event extraction score improves by 4 points (Table 5). This improvement is mainly due to an increase in recall while the precision, more or less, stays the same. However, for the Part-of relation, the recall significantly increases (23% to 45%) while the precision decreases (79% to 66%), resulting in a significantly higher F-score. The localization event recall (Table 6, row 2) is on average 20% lower for all types than the location entity recall (Table 6, row 1), which indicates that the events are equally difficult to predict independently of the argument types. The prediction of hosts and bacteria entities achieved a very good recall of 84 and 82 (row 1), respectively, indicating the relevance of the NCBI resource and additional patterns. Geographical locations were poorly recognized (29%), but the new combination of an appropriate DBpedia dictionary and the contextual patterns greatly improved the recall by 41 points, probably slightly decreasing the entity precision, as shown by the poor event precision of geographical entities (36%). Environment arguments remain harder to predict due to their high naming variability (61%).

The localization event precision (Table 6, row 3) is more difficult to analyze than the recall because the precision of the argument prediction is not measured. Many sources of error may be involved, such as incorrect arguments, incorrect anaphora resolution, relation to the wrong bacterium among several or the lack of a relation.

The investigation of the test corpus also shows that in some cases environmental locations were mentioned in the documents as potential sources of industrial applications without actually being bacteria isolation sites. The Alvis system does not handle such modalities. For instance, in *Some enzymes of Thermus species *[...] *Other fields of application [...], waste treatment, pulp and paper manufacture, and animal feed and human food *(BTID-50167), the Alvis system erroneously relates *Thermus *to *waste treatment*, *paper manufacture*, *animal feed *and *human food*.

The event prediction results denoted by the F-score vary with respect to the type of location (row 4). The best result is obtained for the Soil type (70.3%), followed by Host (59%) and Host-part (46.8%). The lowest F-score is obtained for the Environment type (30.5%), followed by Geographical (33.5%) and Water (39%), which also had the worst entity recall. These results indicate that the recognition of entities varies in difficulty. Most of the soil terms have the word *soil *as their head, making them easier to type. Conversely, the environments are the most unpredictable due to their diversity. Compared with the official results, the new results increased for all of the types, except for Water. This improvement is explained by the refinement of the syntactic analysis and head word identification method, which is critical for location type assignment. The Water exception deserves further investigation. Considering the low number of test examples (21), it may be due to the introduction of a few type errors as a consequence of the MBTO expansion.

### Results by resource

The three lexical resources DTa2, EnvO and MBTO yield different results, as shown in Tables 7 and 8. Using only EnvO yields the worst results, with an F-score drop of approximately 14 points compared to DTa2 (Table 7). This result is due to the small intersection of location term sets in EnvO and in the test corpus. Ambiguous terms and heads in EnvO affect the performance of the symbolic syntactic-semantic typing (see Table 3). The union of DTa2 and MBTO achieves the best F-score (49%) compared with using the two resources independently. MBTO matches more terms than DTa2 (3.8 point increase in recall) but introduces typing ambiguities, as shown by a decrease in precision (Table 7).

**Table 7:** Scores obtained by different resources and the symbolic syntactic-semantic type assignment method.

	Entity recall	Recall	Precision	F-score
DTa2	74.6	47.6	50	48.8
MBTO	78.6	51.4	46	48.5
MBTO+DTa2	79.2	52.1	46.3	49.1
EnvO	63.9	30.4	38.8	34.1

**Table 8:** Scores obtained by different resources and the exact match type assignment method.

	Entity recall	Recall	Precision	F-score
DTa2	64.3	37.7	51.9	43.7
MBTO	69.3	41.3	46.5	43.7
MBTO+DTa2	69.7	41.7	47.1	44.3
EnvO	58.4	28.7	46	35.4

### Experimental results per method

**Figure 3:**
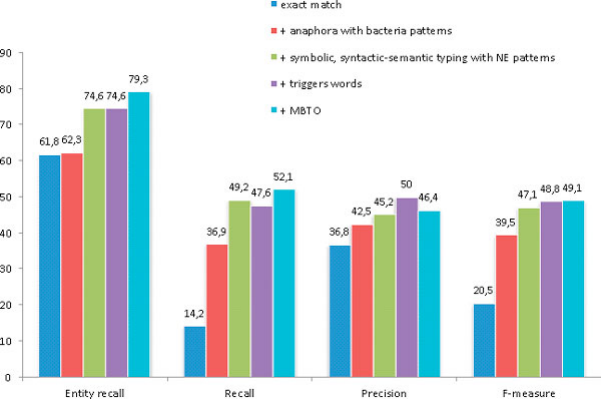
**Score improvement**. Performance changes with the addition of different modules. The baseline performance is the result obtained using only publicly available resources. Each subsequent experiment involves the addition of the specified modules.

Figure 3 displays the cumulative benefit of each component of our approach. The baseline method simply projects publicly available resources, including the NCBI list for bacteria recognition, the DTa2 terms, the NCBI eukaryote tree and the DBpedia country list for the location extraction and typing. The addition of the anaphora resolution algorithm and bacteria name entity patterns increase the score by 19 points, indicating that bacteria argument names are often not explicit and, therefore, require more sophisticated linguistic methods for their retrieval. While the improvement in precision is due to more bacteria being properly extracted using the bacteria patterns, this module mainly increases the recall. The symbolic syntactic-semantic typing strategy improves the score by 7.6 points, indicating that a head-matching method is efficient. This module includes the entity patterns used for the prediction of Bacteria, Host and Geographical types. Moreover, the scores obtained using only DTa2 as a resource suggest that the test set is consistent with the training and development sets. Adding the trigger word constraint for event prediction increases the F-score by only 1.7 points. However, this feature balances out the recall and precision and more precisely defines a localization event by ensuring that a sentence contains a word that signals a localization other than the bacterium and location arguments. The addition of the MBTO ontology together with DTa2 increases the recall and lowers the precision of event prediction, resulting in a 0.3-point increase. This module confirms that using MBTO results in more localizations being predicted but with a lower accuracy.

The symbolic syntactic-semantic algorithm allows us to type previously unobserved terms (an average recall increase of 8 points compared with exact match; see Tables 7 and 8). However, the mapping of terms to heads is not one-to-one, possibly resulting in a term having two or more types. This method, in turn, can introduce some ambiguity, resulting in a lower precision (average precision decrease of 2.5 points; see Tables 7 and 8). Overall, the typing algorithm grants a significant 4.9 % average increase.

For the baseline, the entity recall is rather high (61.8 %), indicating that using well-adapted and complete resources allows us to recover a high proportion of arguments. The symbolic syntactic-semantic typing method also significantly increases the entity recall, demonstrating that the approach we use is effective and relevant for this task. Overall, Figure 3 shows the contribution of each of the strategies presented in this paper. Each of the four modules presented displays an important (recall and/or precision) and significant improvement of the overall score.

## Discussion

Future work will focus on the improvement of the prediction of location entities and events using more syntactic information.

Term extraction associated with the symbolic syntactic-semantic typing method appears to be very efficient for predicting locations, including unobserved entities. The similarity of the training and test set vocabulary resulted in a good performance of the argument prediction using the DTa2 annotated terms. However, MBTO obtained similar results. With the typing method, MBTO should, therefore, demonstrate higher prediction capabilities than DTa2 on new corpora for which no manual annotations are provided.

Regardless of the lexicon richness, the typing strategy excludes all terms with unknown heads, which may be critical in cases where the corpus may not be representative of the available lexicon. In the future, we will overcome this potential limitation by studying the benefit of using linguistic markers, such as exemplification structures, for recovering additional location terms. For example, in the exemplification expression *heated organic materials, such as compost heaps, rotting hay, manure piles or mushroom growth medium*, our method would correctly type not only *heated organic materials *as Environment but also the other location examples of the enumeration despite their unknown heads.

Alvis predicts localization and Part-of events by co-occurrence of the arguments with trigger words within the same sentence. This method has a negative effect on the precision measure because some pairs are irrelevant, as in example 5. Two hosts are predicted for the *Baumannia *bacterium, namely the leafhopper and the plant. However, only the first one is correct.

### 

#### Example 5 (BTID-10075)

***Baumannia cicadellinicola***. *This newly discovered organism is an obligate endosymbiont of the **leafhopper insect Homalodisca coagulata (Say)**, also known as the **Glassy-Winged Sharpshooter**, which feeds on the xylem of **plants***.

It has been shown that the use of syntactic dependencies to extract biological events, such as protein-protein interactions, improves the results of such systems [32,23,20] because syntactic dependencies indicate semantic roles. In example 6, syntactic dependencies would be useful to distinguish the host among the entities by searching for the one that is directly related to the bacteria by an agent - action - target relation. The use of syntactic dependencies could offer a deeper examination of the syntax and semantics, thereby allowing for a more refined extraction of bacteria localization and host-host part relations. However, our preliminary experiments with syntactic parsing and machine learning indicate that the high variability of syntactic structures of corpus sentences may be a significant obstacle for the induction of efficient prediction rules. This idea has yet to be investigated.

## Conclusion

The outcome of this work concerns both the biology and the information extraction domains. The promising performance of the Alvis system on the BB task shows that a combination of semantic analysis and domain-adapted resources is a good strategy for information extraction in the biotope domain. We present an argument prediction method based on term extraction and linguistic analysis of EnvO and MBTO habitat resources. The results obtained using MBTO indicate that our argument prediction method is efficient even without any training data. Its potential application scope should be far beyond the BB task and deserves further evaluation on large collections of scientific papers that are rich in biotope information. We have also shown that the anaphora resolution algorithm considerably improves the event extraction by considering not only usual anaphoric expressions, such as pronouns, but also hyperonyms and cardinality. Definite anaphoric expressions are not specific to the BB corpus and are frequent in technical and scientific documents in experimental domains. The benefit of a combination of the two methods still has to be evaluated in other information extraction domains. A further characterization of the corpus and events for which the method is adapted must also be performed.

## Competing interests

The authors declare that they have no competing interests.

## Authors' contributions

All authors contributed to the production of this manuscript. WG performed the term extraction. ZR conceived and implemented the bacteria name recognition. WG and PW conceived and implemented the term typing algorithm. ZR conceived and implemented the anaphora resolution and relation extraction. All authors read and approved the final manuscript.
